# Psychological support by full‐time school counselors from the City of Nagoya after the 2024 Noto Earthquake: An activity report

**DOI:** 10.1002/pcn5.70074

**Published:** 2025-03-04

**Authors:** Hiromichi Inaba, Yoshitaka Nishikawa, Hiroko Tsuboi, Yukiyo Nagai, Kei Ohashi, Rie Yamada, Masatsugu Sakata, Akane Nogimura, Toshiya Murai, Atsurou Yamada

**Affiliations:** ^1^ Department of Psychiatry Kyoto University Graduate School of Medicine Kyoto Japan; ^2^ Department of Health Informatics Kyoto University School of Public Health Kyoto Japan; ^3^ Graduate School of Humanities and Social Sciences Nagoya City University Nagoya Japan; ^4^ Department of Neurodevelopmental Disorders Nagoya City University Graduate School of Medical Sciences Nagoya Japan

## Abstract

The City of Nagoya employs full‐time school counselors in its schools. This activity report highlights the role of them in offering psychological support after the disaster.

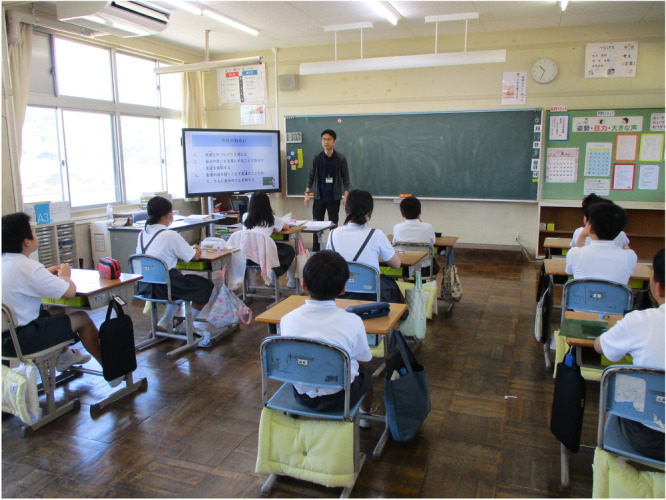

Issues related to children's mental health in Japan, such as suicide, bullying, abuse, and refusal to attend school, are becoming increasingly severe.^1^ Concomitantly, there is a growing interest in children's mental health worldwide, and incorporating mental health services into school settings has been considered a countermeasure.^2^, ^3^ The School Counselor (SC) program was established in Japan in 1995. However, it primarily consists of part‐time positions, with assignments limited to fewer than 10 h per week.^4^ Nagoya is the only city in the country that has implemented a policy since April 2014 to place full‐time SCs and other specialists, such as social workers, in schools. These professionals have formed a team called the “Nagoya Child Advocacy Committee (NCAC)” to support students. Being full‐time allows them to engage with children regularly, enabling the early detection and prevention of mental health problems and providing individual support, thereby encouraging the development of all children. Collaboration with families, communities, and organizations is enhanced. Additionally, the burden on teachers is expected to ease. This activity report highlights the role of the NCAC in offering psychological support after the 2024 Noto Earthquake.

Japan has experienced numerous natural disasters due to its geographical and climatic conditions. After disasters, “public assistance” efforts, such as rescue, lifesaving, personnel dispatch, and material and financial support, are crucial.^5^ On January 1, 2024, a 7.6‐magnitude earthquake struck the Noto Peninsula in Ishikawa Prefecture, resulting in over 400 deaths, destruction of more than 136,000 buildings, and other extensive damage^6^ (Figure 1a).

**Figure 1:**
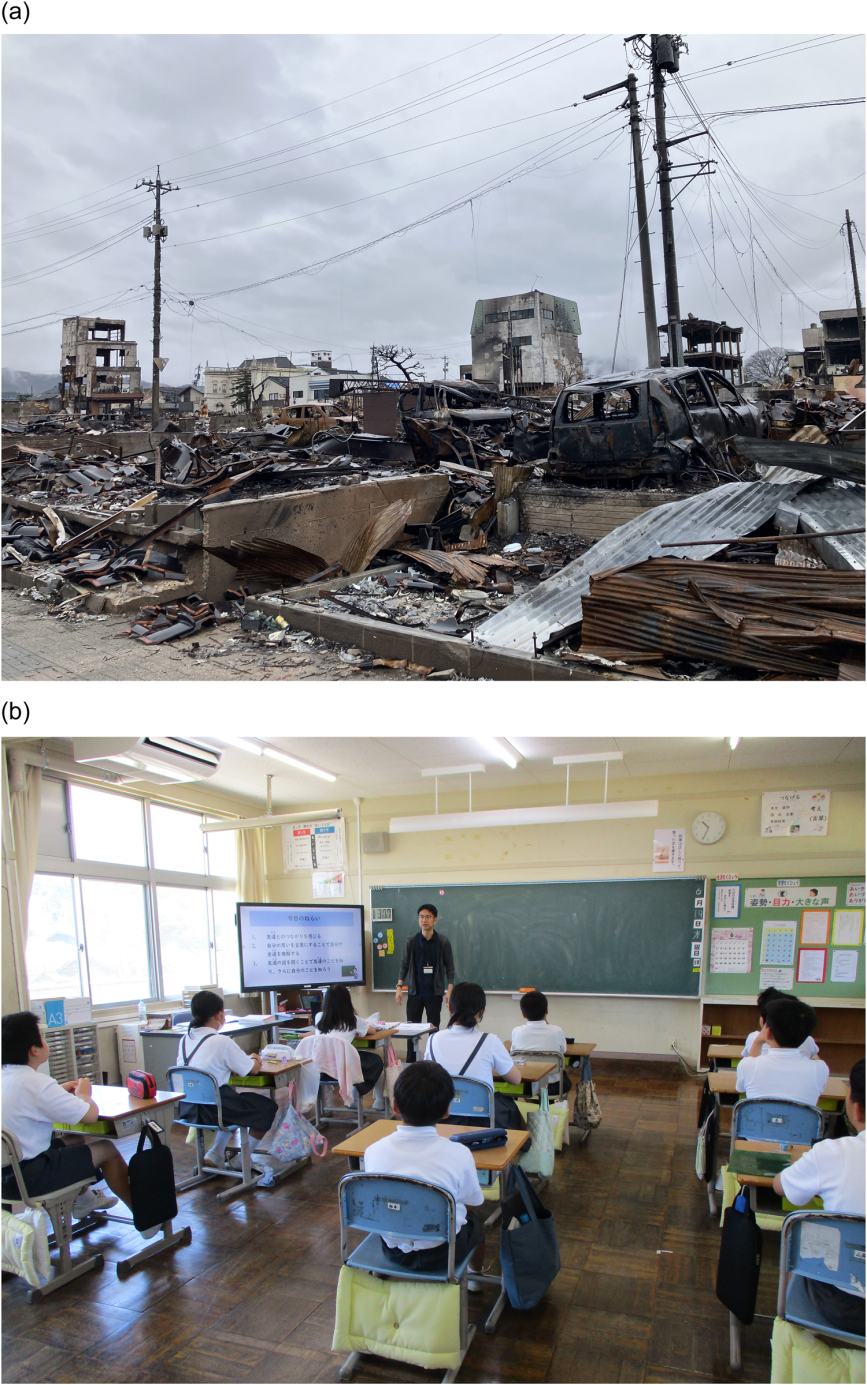
(a) Aftermath of the Noto Peninsula Earthquake in Ishikawa Prefecture on January 1, 2024. (b) Scenes of psychoeducation in disaster‐affected areas.

At the national government's request, in the Aichi Prefecture, where the City of Nagoya is located, local psychology associations took the lead in assembling and dispatching a team of psychologists from within the prefecture. A team of four psychologists was sent each week, with two of the four members always being SCs affiliated with the NCAC. The teams focused on Wajima City, one of the most severely affected areas. These activities commenced in February amid inadequate conditions, such as disrupted transportation networks and damaged infrastructure. They visited reopened schools and interviewed all attending students, accounting for 10% of the total student body. The purpose was to screen as well as to provide psychoeducation (e.g., providing information on how to cope with acute stress reactions). In April, they interviewed students who had not been screened and teaching staff. In addition to the previous activities, interviews with parents, psychoeducation for students and teaching staff, and classroom observations were conducted in May. From June onward, the focus shifted from individual interviews to psychoeducation, staff support, and individual consultations. Regardless of the time or interviewees, great care was taken to ensure that the process did not lead to retraumatization, and the situation was handled cautiously. Interviews were attempted for all students, but none were conducted with those who declined. For teaching staff and parents, interviews were conducted upon request. These activities can be considered as primary prevention activities for mental health problems. In particular, the full‐time SCs of the NCAC were accustomed to primary prevention activities in the school setting, allowing them to operate in the same way as usual (Figure 1b).

There were several advantages of using the full‐time system over the part‐time system previously used for school counselors in the City of Nagoya. First, it is easier to secure personnel, allowing long‐term, stable, and continuous support. Previously, part‐time counselors required permission from multiple supervisors to provide support, making long‐term staffing difficult, especially during the fiscal year transitions in April. However, with support from the NCAC, stable scheduling has been achieved even during this transition. Second, support experience was accumulated within the organization, facilitating information sharing, task handovers, and aftercare among the staff. Returning staff can share their experiences with colleagues, enabling the subsequently dispatched staff to engage in support activities more effectively. These experiences are expected to be beneficial for future disaster relief efforts. Furthermore, disaster relief workers are at an increased risk of developing mental disorders due to occupational trauma exposure.^7^ However, the NCAC staff could receive the necessary peer mental support from supervisors and colleagues at their workplaces even after completing their support activities. Finally, since support personnel are dispatched as employees of the City of Nagoya, the administrative work associated with temporary employment is reduced, which is beneficial for disaster‐affected areas where support acceptance systems can be challenging.^8^ These findings suggest that the City of Nagoya's policy of deploying full‐time specialists in schools, originally intended to strengthen support for children, also has functional advantages in providing psychological support after disasters.

## AUTHOR CONTRIBUTIONS

Hiromichi Inaba, Yoshitaka Nishikawa, and Atsurou Yamada planned the study, while Hiromichi Inaba and Atsurou Yamada conducted interviews with the Nagoya City Board of Education and drafted the manuscript. All authors critically reviewed the manuscript.

## CONFLICT OF INTEREST STATEMENT

Atsurou Yamada is an Editorial Board member of *Psychiatry and Clinical Neurosciences Reports* and a co‐author of this article. To minimize bias, he was excluded from all editorial decision‐making related to the acceptance of this article for publication. Yukiyo Nagai, Kei Ohashi, Rie Yamada, Masatsugu Sakata, Akane Nogimura, and Atsurou Yamada are employed in the Department of Neurodevelopmental Disorders, Nagoya City University Graduate School of Medical Sciences, which is an endowment department supported by the City of Nagoya. Hiroko Tsuboi is an Endowed Course Professor, supported by the City of Nagoya, in the Graduate School of Humanities and Social Sciences, Nagoya City University. Outside the submitted work, Masatsugu Sakata has received a personal fee from SONY; Yoshitaka Nishikawa has received a grant from Japan Medical Association and donations from Datack and Cancerscan; and Toshiya Murai has received speakers honoraria from Takeda Pharmaceutical, Sumitomo Pharma, Kracie Pharmaceutical, Otsuka, Meiji‐Seika Pharma, Janssen Pharmaceutical, Lundbeck, EA Pharma, Nippon Boehringer Ingelheim and Viatris, an editing fee from Meiji‐Seika Pharma, and research funding from Shionogi & Co. Ltd., The Japan Kanji Aptitude Testing Foundation, Bonbon Inc., Dai Nippon Printing, Sumitomo Pharma, Otsuka, and Eisai. The remaining author declares no conflicts of interest.

## ETHICS APPROVAL STATEMENT

This study did not require ethical approval, as it focused on the psychological support after the 2024 Noto Earthquake by full‐time school counselors from the City of Nagoya and did not involve direct interaction with, or data collection from, human participants.

## PATIENT CONSENT STATEMENT

Not applicable.

## CLINICAL TRIAL REGISTRATION

Not applicable.

## Data Availability

Data sharing is not applicable to this article as no datasets were generated or analyzed during the current study.
